# Genomic Regions Associated with Fusarium Wilt Resistance in Flax

**DOI:** 10.3390/ijms222212383

**Published:** 2021-11-17

**Authors:** Alexander Kanapin, Mikhail Bankin, Tatyana Rozhmina, Anastasia Samsonova, Maria Samsonova

**Affiliations:** 1 Centre for Computational Biology, Peter the Great St. Petersburg Polytechnic University, 195251 St. Petersburg, Russia; a.kanapin@gmail.com (A.K.); a.a.samsonova@gmail.com (A.S.); 2 Mathematical Biology & Bioinformatics Laboratory, Peter the Great St. Petersburg Polytechnic University, 195251 St. Petersburg, Russia; mikle.p.bankin@gmail.com; 3 Laboratory of Breeding Technologies, Federal Research Center for Bast Fiber Crops, 172002 Torzhok, Russia; tatyana_rozhmina@mail.ru

**Keywords:** fusarium wilt, resistance, flax, GWAS, immune response, disease severity index

## Abstract

Modern flax cultivars are susceptible to many diseases; arguably, the most economically damaging of these is the Fusarium wilt fungal disease. Over the past decades international flax breeding initiatives resulted in the development of resistant cultivars. However, much remains to be learned about the mechanisms of resistance to Fusarium infection in flax. As a first step to uncover the genetic factors associated with resistance to Fusarium wilt disease, we performed a genome-wide association study (GWAS) using 297 accessions from the collection of the Federal Research Centre of the Bast Fiber Crops, Torzhok, Russia. These genotypes were infected with a highly pathogenic *Fusarium oxysporum* f.sp. *lini* MI39 strain; the wilt symptoms were documented in the course of three successive years. Six different single-locus models implemented in GAPIT3 R package were applied to a selected subset of 72,526 SNPs. A total of 15 QTNs (Quantitative Trait Nucleotides) were detected during at least two years of observation, while eight QTNs were found during all three years of the experiment. Of these, ten QTNs occupied a region of 640 Kb at the start of chromosome 1, while the remaining QTNs mapped to chromosomes 8, 11 and 13. All stable QTNs demonstrate a statistically significant allelic effect across 3 years of the experiment. Importantly, several QTNs spanned regions that harbored genes involved in the pathogen recognition and plant immunity response, including the KIP1-like protein (*Lus10025717*) and NBS-LRR protein (*Lus10025852*). Our results provide novel insights into the genetic architecture of flax resistance to Fusarium wilt and pinpoint potential candidate genes for further in-depth studies.

## 1. Introduction

Flax (*Linum usitatissimum* L.) is a valuable crop cultivated for oil and fiber. One of the major flax pathogens affecting the world’s crop production is the fungus of the genus *Fusarium*, *Fusarium oxysporum* f.sp. *lini*. The primary fungal infection occurs through the roots. Next, the pathogen colonizes the xylem and blocks the flow of water and nutrients causing the yellowing and wilting of the leaves, vascular tissue damage and, ultimately, plant death [1]. As a result of the disease outbreak, 80–100% of flax harvest could be lost. Moreover, the fungus chlamydospores can survive for up to 50 years in the infected soil and are extremely difficult to eliminate [2].

Flax wilt management is achieved through various agricultural practices, pesticides [3,4] and breeding efforts that aim to cultivate resistant crop varieties [5,6,7,8]. Pesticides used in excess and irresponsibly are hazardous for human health [9]. Furthermore, they reduce biodiversity; therefore, they are detrimental to the structure and function of an ecosystem [9,10].

In view of the above, the best approach for effective disease control is a cultivation of resistant genotypes in rotation with other crops. The majority of modern flax varieties show high or moderate resistance towards Fusarium wilt [7,8]. However, there is a significant risk of disease development due to variations of pathotypes in fungal populations and a substantial genetic erosion of varietal material. This situation is further aggravated by global warming. Climate change and other natural, human-induced stressors, may lead to a rise in aggressiveness of individual pathogen races, as well as to the loss of resistance by the varieties determined by one or two genes. Therefore, it is of utmost importance, to take proactive steps, and to create varieties with different effective pathogen-resistant genes, as well as with optimal combinations of these genes to ensure long-term and efficient crop protection.

The mechanisms of flax resistance to Fusarium wilt have never been fully understood, although resistance to the disease was developed by selection and recombination [7,8]. The cornerstone of a plant’s immune system is represented by two classes of receptors that recognize pathogens and protect plant tissues against fungal invasions [11]. At the initial stage of the plant–pathogen interaction, pattern recognition receptors (PRRs) play an important role in pathogen perception and early immune response. All well-characterized PRRs are evolutionary, conserved, membrane-localized, receptor-like kinases. They trigger the PAMP, i.e., pattern-triggered immunity (PTI) by recognizing pathogen-/microbial-associated molecular patterns. PTI plays important role in basal resistance that generally develops after the successful infection of an adapted pathogen [12]. The second class of receptors involved in the establishment of the next level of defense, i.e., the effector-triggered immunity (ETI) is activated when a plant R-gene protein recognizes an effector encoded by the avirulence (*Avr*) gene of the pathogen [13,14]. R genes belong to five classes formed by genes encoding nucleotide-binding domain, leucine-rich, repeat-containing (NBS-LRR) proteins, receptor-like kinase genes, genes producing receptor-like transmembrane proteins or serine threonine kinases, and atypical R genes [15]. To date, over 300 R genes have been characterized and cloned. Of these, more than 60% contain NBS and LRR domains [16,17]. Both PTI- and ETI-resistant regulatory cascades overlap in downstream signaling pathways, which include MAP kinase (MAPK) cascades, calcium fluxes, reactive oxygen species (ROS) production and alterations in hormone networks [18]. Pathogen-related proteins, components of the cell wall, transcription factors, secondary metabolites and antioxidants all play essential roles in the response of flax to the *F. oxysporum* f.sp. *lini* infection, as demonstrated by several transcriptomics experiments [19,20,21].

Identification of resistance genes and quantitative trait loci are key elements of a successful breeding program. Due to its increased economic importance in the past couple of decades flax has become a centre spot of attention and an object of intensive research in the field of genomics. Recently numerous GWAS studies carried out in flax which interrogated traits related to yield, phenology and fatty acid content were reported [22,23,24,25,26,27,28,29]. In addition, flax omics data analytics broadened our knowledge of wilt resistance genes obtained with classical genetics approaches by adding data on QTL loci to a known gene set (~10 genes) conferring wilt resistance in flax [7,8,30]. Specifically, Spielmeyer et al. [31,32] identified two QTLs explaining 38% and 26% of phenotypic variations, located within linkage groups 6 and 10. These loci were discovered in a population of double haploid lines developed from crosses between flax wilt-resistant and susceptible parents. Overall, this plethora of information could be further used in follow-up experiments to decipher resistance mechanisms [33] or in breeding programs for the development of superior cultivars [34].

In this study we aim to exploit flax natural variations to uncover genomic regions controlling resistance to Fusarium wilt. We performed the whole-genome sequencing of 297 flax accessions at 10× depth coverage to characterize genetic diversity and population structure specific to the most Russian varieties [35]. We evaluated the accessions for disease resistance under controlled conditions and applied GWAS algorithms which relied on different statistical models to reveal SNP trait associations. To our knowledge, this is the first time that the GWAS approach was applied to interrogate Fusarium wilt resistance in flax.

## 2. Results

### 2.1. Evaluation Resistance to Fusarium Wilt in Flax

**Figure 1:**
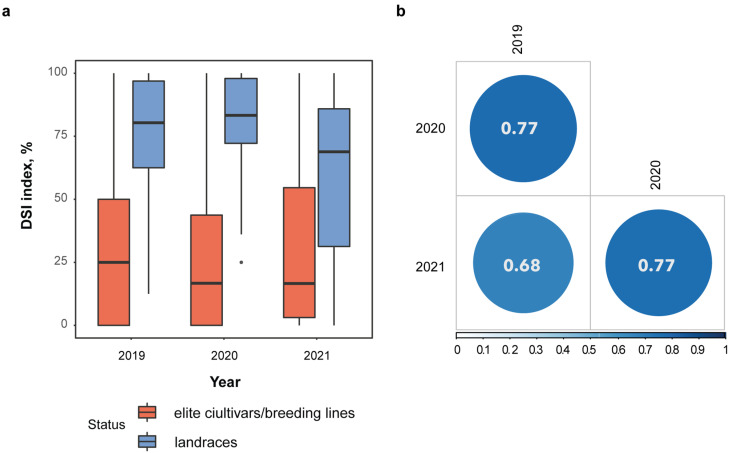
Fusarium wilt resistance in the dataset. (**a**) DSI index values in elite cultivars/breeding lines and landraces. Black dots represent outlier values, (**b**) The between year correlation of DSI index.

Flax resistance to highly virulent *F. oxysporum* MI39 was evaluated under greenhouse conditions by calculating the Disease Severity Index (DSI) (Table S1). The DSI is a normalized proportion of genotypes with identical disease symptoms (see Section 4). The accessions were classified as resistant (42%), weakly susceptible (7%), moderately susceptible (17%) and susceptible (34%) using average DSI values, across 3 years. DSI values are strongly correlated across all three years of investigation (Figure 1), thus attesting to the consistency of the phenotypic dataset. The DSI values obtained for flax cultivars and breeding lines significantly differ from landraces (*p*-values for 2019–2021 years of experiment were 7.1 × 10^−6^, 1.96 × 10^−7^ and 0.52 × 10^−3^, correspondingly) which may be indicative of breeding efforts aimed at the development of wilt-resistant genotypes (Figure 1).

### 2.2. Association Mapping of Fusarium Wilt Resistance

A total of 72,526 variants retained after SNP calling and filtering (thresholds for MAF (Minor Allelle Frequency) and call rate were 0.05 and 0.85, respectively) were widely distributed over 15 flax chromosomes (Figure S1a). Patterns of population differentiation were analyzed using a principal components analysis, which did not produce a clear separation of fiber and linseed genotypes (see bi-plot in Figure S1b,c).

The precision of the genetic association test depends on the scale of LD (Linkage Disequilibrium), which implicitly defines the size of marker set required for analysis. With a genome size of about 370 Mb and the mean LD decay of 8.6 Kb [35], the number of markers in our dataset (i.e., 72,526 loci) was twice the minimal number of SNPs necessary for an accurate genetic test.

Genome-wide association analyses were performed using the following models: GLM, MLM, CMLM, FarmCPU, SUPER, Blink as implemented in GAPIT3 R package [36]. Using both the GAPIT estimation of Bayesian information content and scree plot analysis, (Figure S1b) we retained a total of five principal components to be used in GWAS.

**Figure 2:**
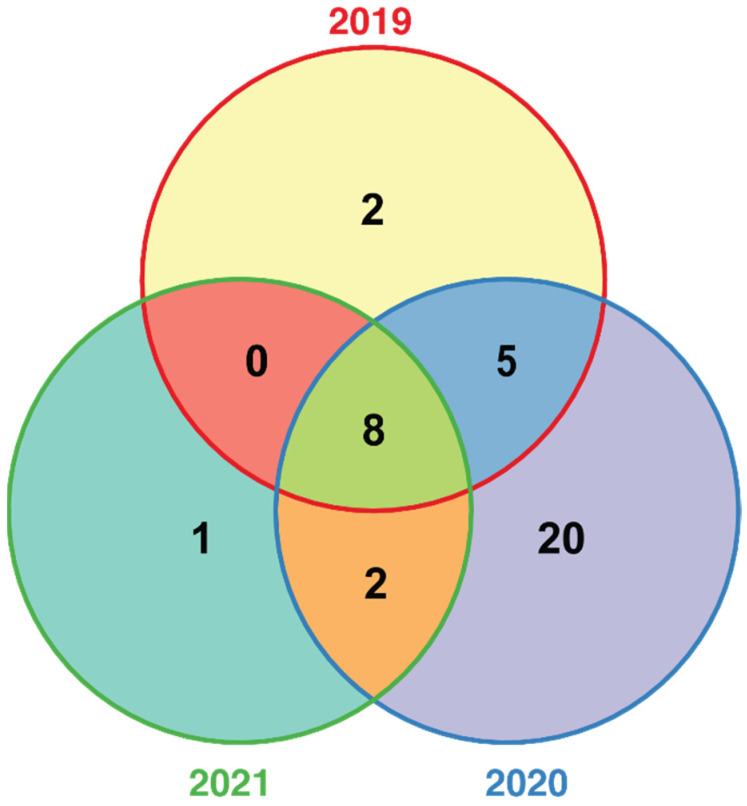
Venn diagram representing the number of unique and shared QTNs associated with DSI index in between-year comparisons.

**Figure 3:**
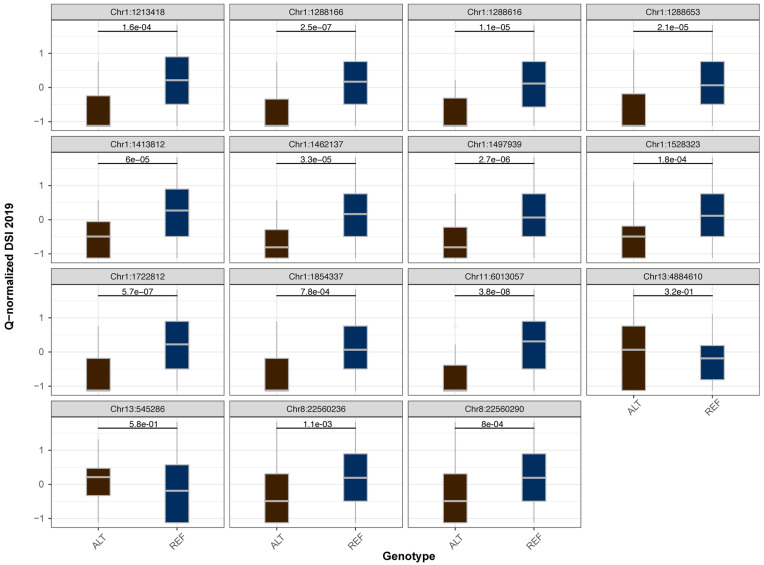
Box plots of the allelic effects observed in stable QTNs in 2019 year. Numbers are *p*-values according to the Mann–Whitney–Wilcoxon non-parametric test.

The six aforementioned methods yielded 38 QTNs identified throughout the experiment (FDR adjusted *p*-value 0.05, see Table S2). All models performed well in controlling population and family structures, as confirmed by respective Q–Q plots where the observed *p*-values deviated from the expected values at the end of distribution. Most of the QTNs with the exception of Chr8:22560236, Chr11:6013057 and Chr13:4884610 were discovered by several models. Fifteen QTNs were detected in at least two years (Table 1), while eight QTNs were found in all years (Figure 2). Importantly, ten of these stable QTNs occupy a region of 640 Kb in length at the beginning of chromosome 1 and explain about 7–10% of the phenotypic variation for resistance to Fusarium wilt. The rest of QTNs are located on chromosomes 8, 11 and 13. The majority of the identified QTNs demonstrate a significant allelic effect across all 3 years (Figure 3 and Figures S2 and S3).

**Figure 4:**
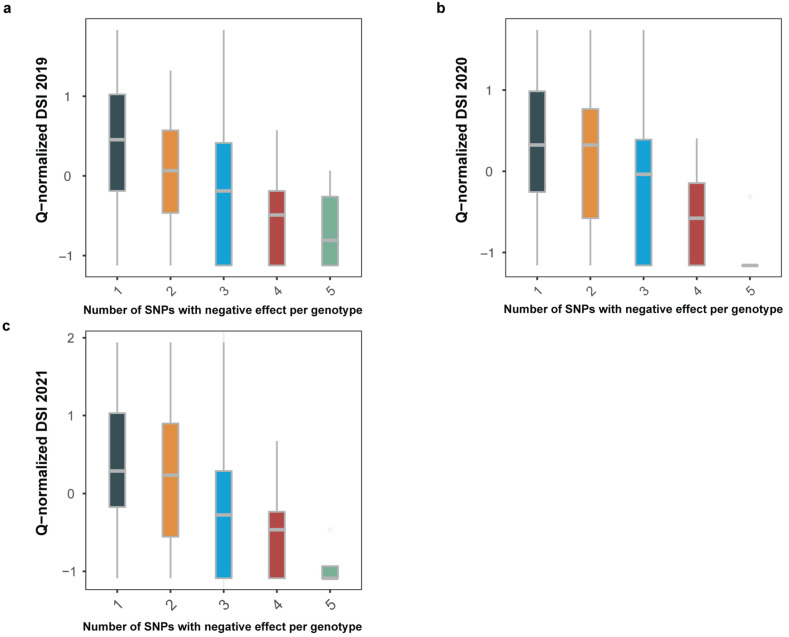
The DSI index value in accessions with different number of QTNs with negative-effect alleles across years of experiment, namely (**a**) year 2019, (**b**) year 2020, and (**c**) year 2021.

Negative alleles decrease the DSI index, while positive alleles increase it. The DSI index value progressively decreases with the increase in the negative-effect allele number in accession genotypes (see Figure 4), thus reflecting a stronger resistance to the pathogen.

### 2.3. Fusarium Wilt Resistance Candidate Genes

Candidate genes involved in various processes associated with pathogen response were identified within predefined regions with length adapted to the extent of LD decay for a relevant chromosome (Figure S4) and centered on stable QTNs (Table 1 and Table 2). Many QTNs detected in this study are orthologous to well-characterized *Arabidopsis* genes. For instance, Chr1:1288653 QTN is a missense variant of *Lus10025717* gene orthologous to *A. thaliana’s AT2G22560* gene, which encodes the KIP1-like protein harboring the actin binding domain. Likewise, QTN Chr1:1462137 is an upstream variant of *Lus10025756* gene, whose *Arabidopsis* ortholog encodes cytochrome P450 monooxygenase from the subfamily CYP709B. Additionally, a missense variant Chr1:1528323 QTN is within the *Lus10025773* gene, which is an ortholog of the *AT1G53050* gene for protein kinase superfamily protein. Chr1:1722812 QTN is an intron variant within *Lus10025823* gene orthologous to the *AT1G79750* gene encoding NADP-malic enzyme 4. Finally, Chr1:1854337 QTN is an upstream transcript variant of *Lus10025853* gene, where the *A. thaliana* ortholog encodes exportin 1A. About 2.5 kb form this QTN is *Lus10025852* gene, where an ortholog in *Arabidopsis* (*AT5G17890*) encodes the DA1-related protein.

Chromosome 8 contains Chr8:22560236 QTN, which is an upstream transcript variant of the *Lus10015356* gene orthologous to the *A. thaliana* HIR1 gene encoding a hypersensitive induced reaction protein. Upstream of this QTN, at a distance of 5.5 kb is the *Lus10015354* gene orthologous to *AT5G40010* gene encoding AAA-ATPase 1. Downstream of Chr8:22560236 QTN are two genes, *Lus10015357* and *Lus10015359*. The closest gene (2.5 Kb), is the ortholog of the *AT5G62740* gene encoding the voltage-dependent anion channel (VDAC) 1 protein, while the other gene, which is 9.4 Kb further downstream, is orthologous to the *AT3G01270* gene for pectate lyase family protein. The *Lus10035917* gene with the Chr11:6013057 QTN is located in the intron of an ortholog of *AT1G80230* gene for rubredoxin-like superfamily protein (Table 2). On chromosome 13, two candidate genes were detected. The *Lus10009330* encoding receptor-like kinase is 1.2 Kb downstream of QTN Chr13:4884610 and is the ortholog of the *A.thaliana RLP12* gene. The second proposed candidate gene, *Lus10009332*, is the ortholog of *AT5G66880* encoding sucrose nonfermenting 1 (SNF1)-related protein.

## 3. Discussion

In contrast to flax rust which, being a model system for H.H. Flor’s series of elegant experiments and a basis for the development of gene-for-gene theory [37], was extensively studied in the past, flax wilt, as well as mechanisms leading to the infection, currently remain unknown.

Recently, several high-throughput transcriptomics experiments provided a general insight into flax resistance to pathogens [19,20,21]. However, the identification of potential key candidate genes in the control of resistance is challenging due to a systemic effect inflicted by infection and, consequently, an unfeasibly large number of genes to be interrogated in focused follow-up studies. To gain an ultimate understanding of biology and mechanisms underlying flax wilt disease, and to prioritize targets for further in-depth experiments, it is essential to make the most of the available genomic and genetic resources, as well as the analytical approaches such as GWAS.

To gain insight into the mechanisms of Fusarium wilt resistance in flax we applied GWAS methods to a collection of 297 flax genotypes which encompassed elite cultivars, breeding lines and landraces. Importantly, the fiber morphotype dominated over the linseed by fifty percent. The disease resistance was estimated by means of the Disease Severity Index (DSI). The DSI shows the normalized proportion of specimens with identical disease symptoms. The genotypes analyzed in this study exhibited a wide variation in resistance to the highly virulent MI39 *F. oxysporum* f.sp *lini* strain in all three successive years of the experiment, thus providing a solid basis for dissecting the genetic architecture of the trait. As expected, elite cultivar and breeding line accessions demonstrated a more than three-fold decrease in DSI as compared to the genotypes with a different selection status, thereby acknowledging the overall success of various breeding programs.

The accurate deciphering of the genetic architecture of a trait in a diverse population is only possible if a statistical model accounts for the spurious associations arising from population structure and family relatedness. Currently, the most popular approach to control for false positives is the incorporation of the population structure and a kinship matrix, which captures family relatedness as covariates in the mixed linear models (MLM). However, all of the MLMs developed to date are single-locus models testing one marker at a time. Such approaches are one-dimensional approximations of a true genetic model of a complex trait controlled by many loci simultaneously and, therefore, they could be inaccurate in controlling false positive associations and estimations of marker effects [38]. To cope with this problem, several single-locus models are generally applied and the associations detected by several models are deemed as significant. We performed association analyses for every year of trait evaluation with six different models: GLM, MLM, CMLM, FarmCPU, SUPER, Blink, as implemented in GAPIT R package [26].

The functional annotation of candidate genes was inferred from their *Arabidopsis* orthologs annotated in the TAIR database [39], as well as from the function of the orthologous genes in other plant species as reported in the literature (Table 2).

The plant response to a pathogen attack involves several tiers of defense, including pathogen sensing by receptor-like kinases (RLK) and R-gene proteins followed by downstream signaling via MAP-kinase cascades, G-proteins and calcium fluxes, which leads to activation of defense and secondary metabolite genes, as well as to the rewiring of hormonal networks. Changes in the calcium status of the cells trigger an oxidative burst. The accumulation of reactive oxygen species (ROS) leads to a hypersensitive response, cell wall protein cross-linking, phytoalexin production, callose deposition and systemic acquired resistance [12,16]. The 640 Kb region on chromosome 1 contains several genes potentially involved in *Fusarium oxysporum* recognition and plant immunity responses. Of these, the *Lus10022557* gene encodes the KIP1-like protein and contains three significant SNPs singled out by the largest negative effects (Table 1). The KIP1-like proteins bind actin [40] and may relay immune signals to inactivate pathogens and interact with RLKs. Therefore, we consider this gene as the most promising candidate for further in-depth downstream analysis [41].

Other potentially promising candidate genes encode the NBS-LRR protein (*Lus10025852*), protein kinase superfamily protein (*Lus10025773*), exportin A1 (*Lus10025853*), NADP-malic enzyme 4 (*Lus10025823*) and cytochrome P450 monooxygenase from CYP709B subfamily (*Lus10025756*). These five proteins are known as significant players in pathogen responses in other plants. First, protein kinases play a central role in signalling during pathogen recognition and the subsequent activation of plant defence mechanisms [42]. The second protein, exportin Xpo1 in *N. benthamiana* is required for elicitor-induced phytoalexin production and the induction of cell death [43]. The third protein, NADP-malic enzyme (NADP-ME), provides the building blocks and energy for the synthesis of two defence-related secondary metabolites, flavonoids and the lignin precursor, monolignol. Moreover, NADP-ME can produce NADPH for synthesis of ROS [44]. Finally, cytochrome P450 monooxygenases from subfamily CYP709B play important roles in plant defence through their involvement in biosynthesis of phytoalexin and some other secondary metabolites [45].

On chromosome 8, we identified four candidate genes in the vicinity of Chr8:22560236 QTN. Two of them, *Lus10015356* and *Lus10015357*, encode a hypersensitive induced reaction (HIR) protein and voltage-dependent anion channel (VDAC) 1 protein, respectively. These genes are likely to be involved in programmed cell death induced by a pathogen attack [46,47,48]. Of the two other genes, *Lus10015354* encodes AAA-ATPase 1, which plays an important role in the salicylic acid-mediated defence response against the blast fungus *Magnaporthe oryzae* [49] in rice. Finally, *Lus10015359* encodes the pectate lyase superfamily protein. This enzyme degrades pectin, a major component of the plant cell wall. During infection, pathogens not only secrete pectin-degrading enzymes, but also hijack the host signalling pathways to induce cell remodelling by plant-derived enzymes [50].

*Lus10035917*, a candidate gene containing Chr11:6013057 QTN, encodes rubredoxin-like superfamily protein (Table 2). In plants, it regulates the reactive oxygen species balance. As a result of the plant response to a pathogen attack and abiotic stresses their levels are increased [51,52,53].

Two candidate genes, *Lus10009330* and *Lus10009332,* were detected downstream of Chr13:4884610 QTN. *Lus10009330* encodes the receptor-like kinase. RLp12, the *A. thaliana* ortholog of this gene, encodes the CLAVATA2-relted protein implicated in innate immunity to microbe and nematode infections [54]. The other gene, *Lus10009332* encodes the sucrose nonfermenting 1 (SNF1)-related protein kinase 2.3. (SnRK). SnRK1 regulates carbon metabolism and responds to hormonal signals. In soybean, SnRK promotes resistance to oomycete *Phytophthora sojae* potentially working through the accumulation of salicylic acid (SA) and induction of SA-related genes [55].

Overall, the candidate genes we pinpointed are involved in pathogen recognition, downstream signaling and plant immunity response and, as such, provide excellent targets for further downstream analyses.

## 4. Materials and Methods

### 4.1. Plant Material Collection and Phenotyping

A total of 297 flax genotypes were selected from the collection of the Federal Research Center for Bast Fiber Crops (Torzhok, Russia) (Table S1). The dataset included 179 fiber flax accessions, 117 linseed flax accessions and 1 accession of unknown morphotype. Oilseed group was further subdivided into the following subgroups: 98 intermediate accessions, 4 large-seeded accessions and 15 crown accessions. There were landraces, elite cultivars and breeding lines in the dataset.

The accessions were evaluated for *Fusarium oxysporum* resistance under greenhouse conditions in randomized complete block design. Three evaluations were performed during 2019–2021. In each evaluation, each specimen was replicated 16 times by sowing all the seeds in cross-container rows. The container dimensions were 550 × 85 × 20 cm. Two genotypes, AP5 and I-7, were used as susceptible and resistant genotypes to Fusarium wilt controls. The infection background was created by introducing 400 g of a pure culture of MI39 *Fusarium oxysporum* f.sp. *lini* strain into a container. The seeds were planted on a 12 day after the inoculation with the pure fungal culture.

The pure culture inoculum was prepared by first growing MI39 on beer-wort agar-agar medium with a subsequent incubation on the oat grain substrate (the grain to water ratio of 1 to 1.75) for 3–4 weeks; such a time period was sufficient for macro and microconidia development. After three to four weeks, when oats were completely infected by the fungus, the pathogen was introduced into the soil. The required amount of the introduced infection for a container (550 × 85 × 20 cm) was established experimentally. The indicator of the reliability of the infectious background was the standard varieties (resistant and susceptible genotypes), which were sown along the edges and in the middle of each container (16 seeds per row).

The evaluation of the disease severity was carried out during the harvesting period in the phase of early yellow ripeness. The DSS (Disease Severity score) grades ranged from 0 to 3, where the 0 value stood for a healthy plant, 1 indicated a partial plant browning or stem browning from one side, 2 indicated a fully browned plant with bolls, and, finally, 3 corresponded to a fully browned plant that collapsed prior to the formation of bolls. Proceeding from these grades the Disease Severity index (DSI) was calculated using the standard formula accepted in phytopathology [56]:

$$\mathrm{DSI}=\frac{\Sigma ab}{AK}\;100\%,\;$$

where *a* is the number of plants with identical DSS, *b* is the estimated DSS; *A* is the total number of plants and *K* is the highest DDS grade (i.e., 3).

The plants were considered resistant if DSI ≤ 20%, as weakly susceptible in the case of 20% < DSI ≤ 30%, as moderately susceptible if 30% < DSI ≤ 50% and, finally, as completely susceptible when DSI value exceeded 50%. The phenotype values were quantile normalized prior to GWAS analyses.

### 4.2. DNA Sequencing and Variant Calling

DNA was extracted from collected leaves with DNeasy Plant Mini Kit (Qiagen, Stanford, CA, USA). DNA sequencing was performed at the BGI (Hong Kong, China) using Illumina protocol, generating paired-end reads 150 bp in length. 1143.850625 GB of raw data comprising 7.626 billon reads with an average of 9.3× coverage or 3.7 Gbp per sample were generated. Processed reads were aligned to NCBI flax reference genome assembly ASM22429v2 with bwa-mem using default parameters [57]. Variant calling was run using NGSEP [58] (v. 4.0) and identified 3416829 biallelic SNPs, which were further filtered using VCFtools [59] to obtain a minor allele frequency (i.e., MAF) > 5% and genotype call rate >85%. A total of 72,526 SNPs passed all filters and remained for further analysis.

### 4.3. Genetic Data Analyses

Principal component analysis (PCA) was conducted using the PCA tools R package [60]. SNP depth and distribution across chromosomes was plotted using rMVP package [61].

The linkage disequilibrium (LD) decay was evaluated using squared Pearson’s correlation coefficient (r^2^). The PopLDdecay [62] version 3.4.1 was run to calculate r^2^ in a 500 kb window. The LD decay was calculated based on r^2^ and the distance for each pair of SNPs using an R script in accordance with Hill–Weir approximation [63].

We applied Mann–Whitney–Wilcoxon test [64] to make group comparisons.

### 4.4. GWAS

The genome-wide association analyses were performed with GAPIT3 R package [36]. We selected the following models: GLM, MLM, CMLM, FarmCPU, SUPER, Blink according to the workflow proposed by the package authors for multiple models testing. The FDR adjusted *p*-value threshold for candidate QTNs selection was set at 0.05.

### 4.5. Candidate Genes

A function of the candidate genes containing the identified QTNs was inferred from the function of their *Arabidopsis* orthologs in the TAIR database, from flax genome annotations kindly provided by Cloutier group (Ottawa Research and Development Centre, Canada) [65], as well as from the function of the homologous genes in other plant species, as described in the literature. We also searched for candidate genes within a window surrounding detected QTNs, where width was defined as proceeding from the LD decay estimated for each chromosome (Figure S4).

## Figures and Tables

**Table 1:** List of stable QTNs identified by several models and in at least two years.

Chromosome		QTN Position	REF/ALT	Average PVE	Average Effect	Year	MAF	QTN Annotation
ID	Number							
CP027619.1	1	1213418	C/T	0.106	−0.53	2019 2020 2021	0.138	upstream_transcript_variant
CP027619.1	1	1288166	A/G	0.09	−0.74	2019 2020 2021	0.093	synonymous_variant
CP027619.1	1	1288616	C/A	0.10	−0.72	2019 2020 2021	0.112	synonymous_variant
CP027619.1	1	1288653	T/C	0.103	−0.76	2019 2020 2021	0.103	missense_variant
CP027619.1	1	1413812	T/A	0.102	−0.69	2019 2020 2021	0.131	intergenic_variant
CP027619.1	1	1462137	A/G	0.082	−0.66	2019 2020	0.099	upstream_transcript_variant
CP027619.1	1	1497939	G/C	0.075	−0.62	2019 2020	0.103	intergenic_variant
CP027619.1	1	1528323	C/G	0.078	−0.64	2019 2020	0.101	missense_variant
CP027619.1	1	1722812	C/G	0.105	−0.68	2019 2020 2021	0.131	intron_variant
CP027619.1	1	1854337	T/C	0.082	0.56	2019 2020	0.101	upstream_transcript_variant
CP027632.1	8	22560236	G/A	0.064	−0.53	2019 2020	0.185	upstream_transcript_variant
CP027632.1	8	22560290	C/A	0.077	0.50	2019 2020 2021	0.19	synonymous_variant
CP027621.1	11	6013057	A/G	0.097	−0.64	2019 2020 2021	0.144	intron_variant
CP027623	13	545286	A/G	0.07	0.52	2020 2021	0.127	intergenic_variant
CP027623	13	4884610	T/G	0.067	1.35	2020 2021	0.457	intergenic_variant

**Table 2:** Candidate genes located near stable QTNs.

QTN	Candidate Gene	Position	*A. thaliana* Orthologue	Protein
Chr11:288653	*Lus10025717*	Chr1:1285554-1289474	*AT2G22560*	KIP1-like protein
Chr1:1462137	*Lus10025756*	Chr1:1463118-1465114	*AT2G46950*, *CYP709B2*	Cytochrome P450, family 709, subfamily B, polypeptide 2
Chr1:1528323	*Lus10025773*	Chr1:1527916-1531752	*AT1G53050*	Protein kinase superfamily protein
Chr1:1722812	*Lus10025823*	Chr1:1722251-1726433	*AT1G79750, NADP-ME4*	NADP-malic enzyme 4
Chr1:1854337	*Lus10025852*	Chr1:1851791-1853596	*AT5G17890, CHS3, DAR4*	DA1-related protein 4, nucleotide-binding, leucine-rich repeat protein
Chr1:1854337	*Lus10025853*	Chr1:1854958-1870647	*AT5G17020, XPO1A*	Exportin 1A
Chr8:22560236	*Lus10015356*	Chr8:22561110-22562338	*AT5G62740, HIR1*	SPFH/Band 7/PHB domain-containing, membrane-associated protein family protein
Chr8:22560236	*Lus10015357*	Chr8:22562785-22565427	*AT5G62740, VDAC1*	Voltage-dependent anion channel 1
Chr8:22560236	*Lus10015344*	Chr8:22554684-22556269	*AT5G40010, AATP1*	AAA-ATPase 1
Chr8:22560236	*Lus10015339*	Chr8:22569641-22570539	*AT3G01270*	Pectate lyase family protein
Chr11:6013057	*Lus10035917*	Chr11:6011724-6014460	*AT1G80230*	Rubredoxin-like superfamily protein
Chr13:4884610	*Lus10009330*	Chr13:4886276-4888681	*AT1G71400, RLP12*	Receptor-like protein 12, RLK
Chr13:4884610	*Lus10009332*	Chr13:4890865-4893541	*AT5G66880, SNRK2.3*	Sucrose nonfermenting 1(SNF1)-related protein kinase 2.3

## Data Availability

The data that support the findings of this study are openly available at European Variation Archive (EVA) Project: PRJEB46073 Analyses: ERZ2775743 (genetic variants).

## Supplementary Materials

The following are available online at https://www.mdpi.com/article/10.3390/ijms222212383/s1.
