# Supplementary figures and images for: Genomic Regions Associated with Fusarium Wilt Resistance in Flax
Source: Int J Mol Sci. 2021 Nov 17;22(22):12383. doi: 10.3390/ijms222212383 (PMC8623186; doi:10.3390/ijms222212383)

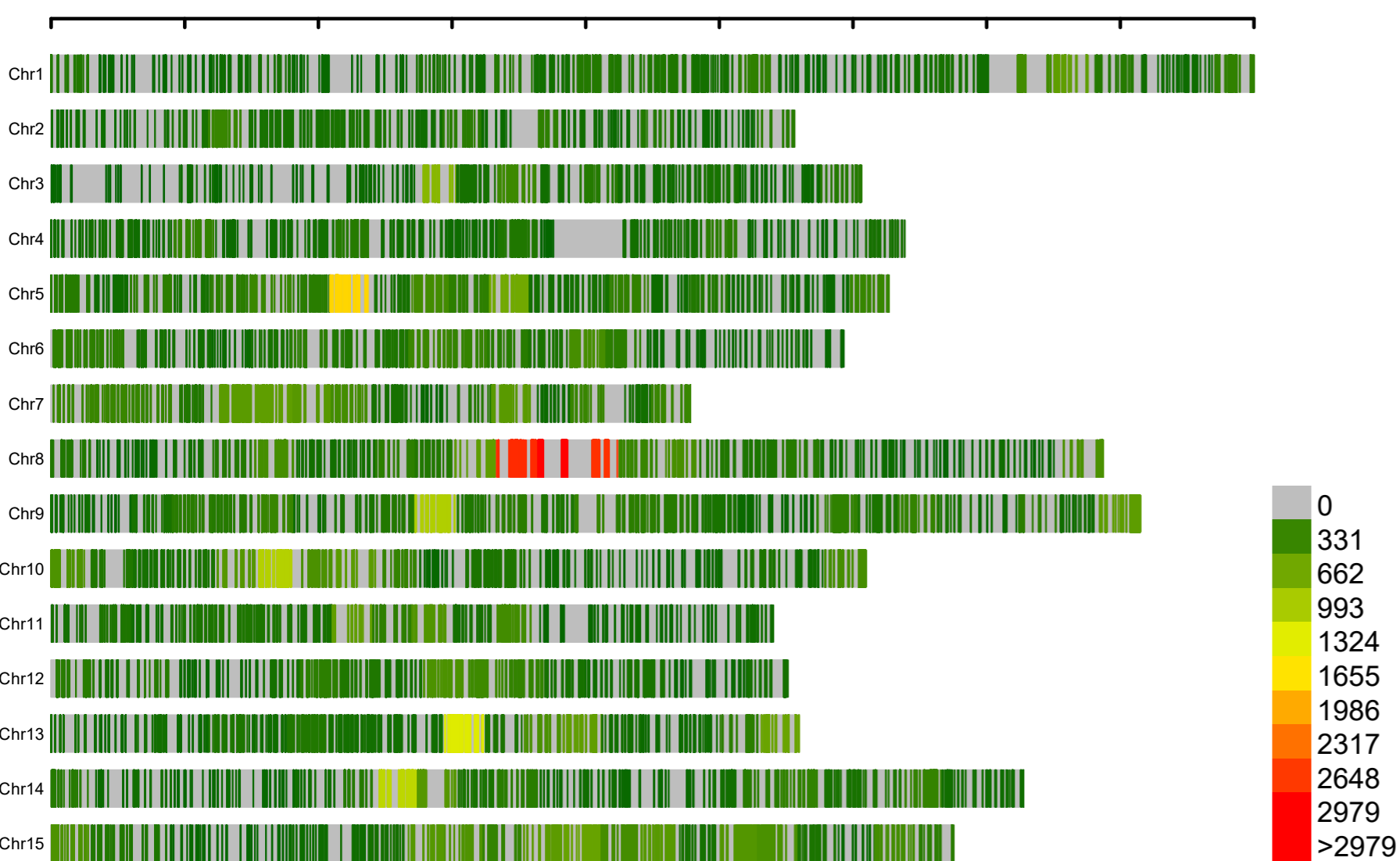

b)

## SCREE plot

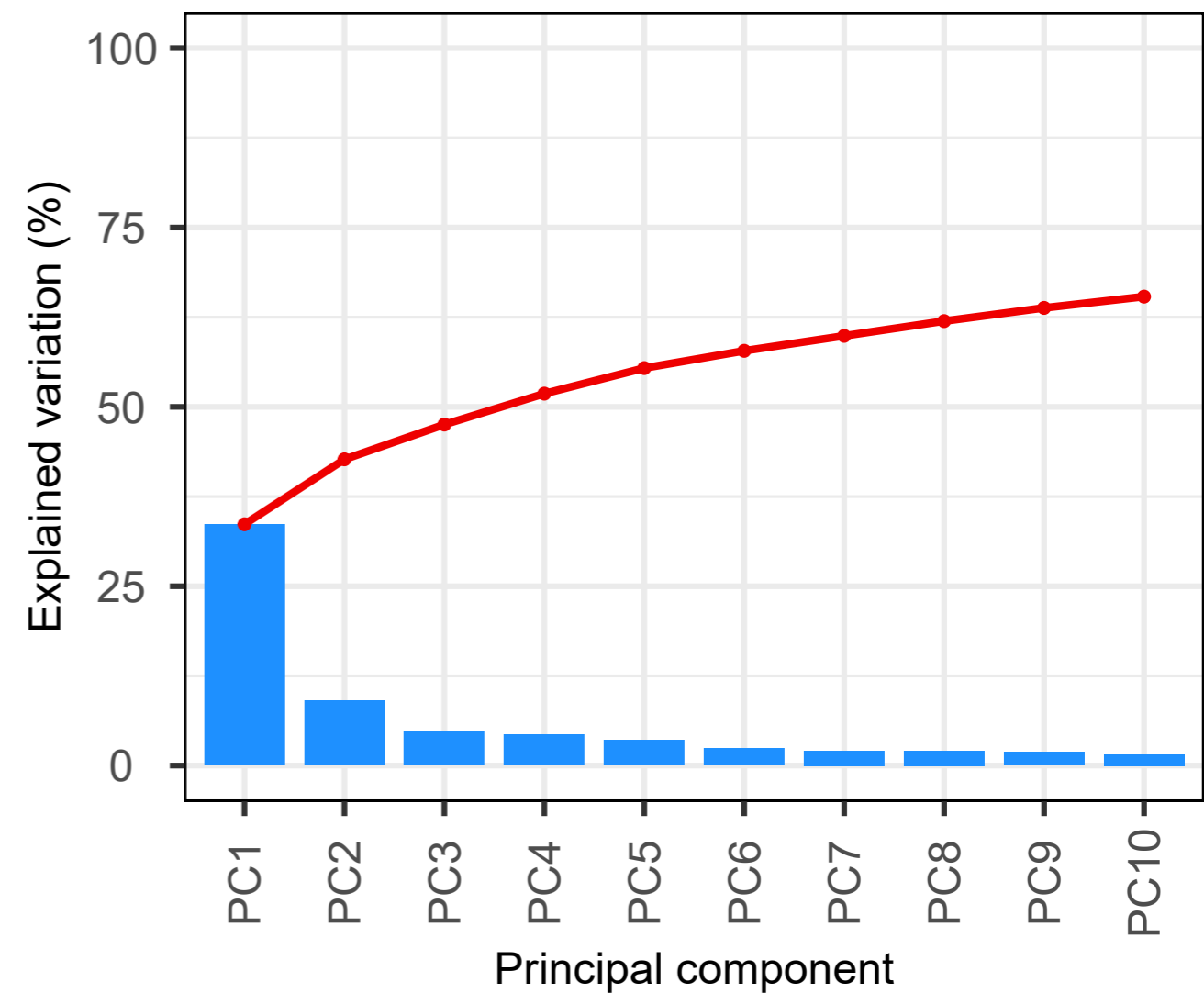

c)

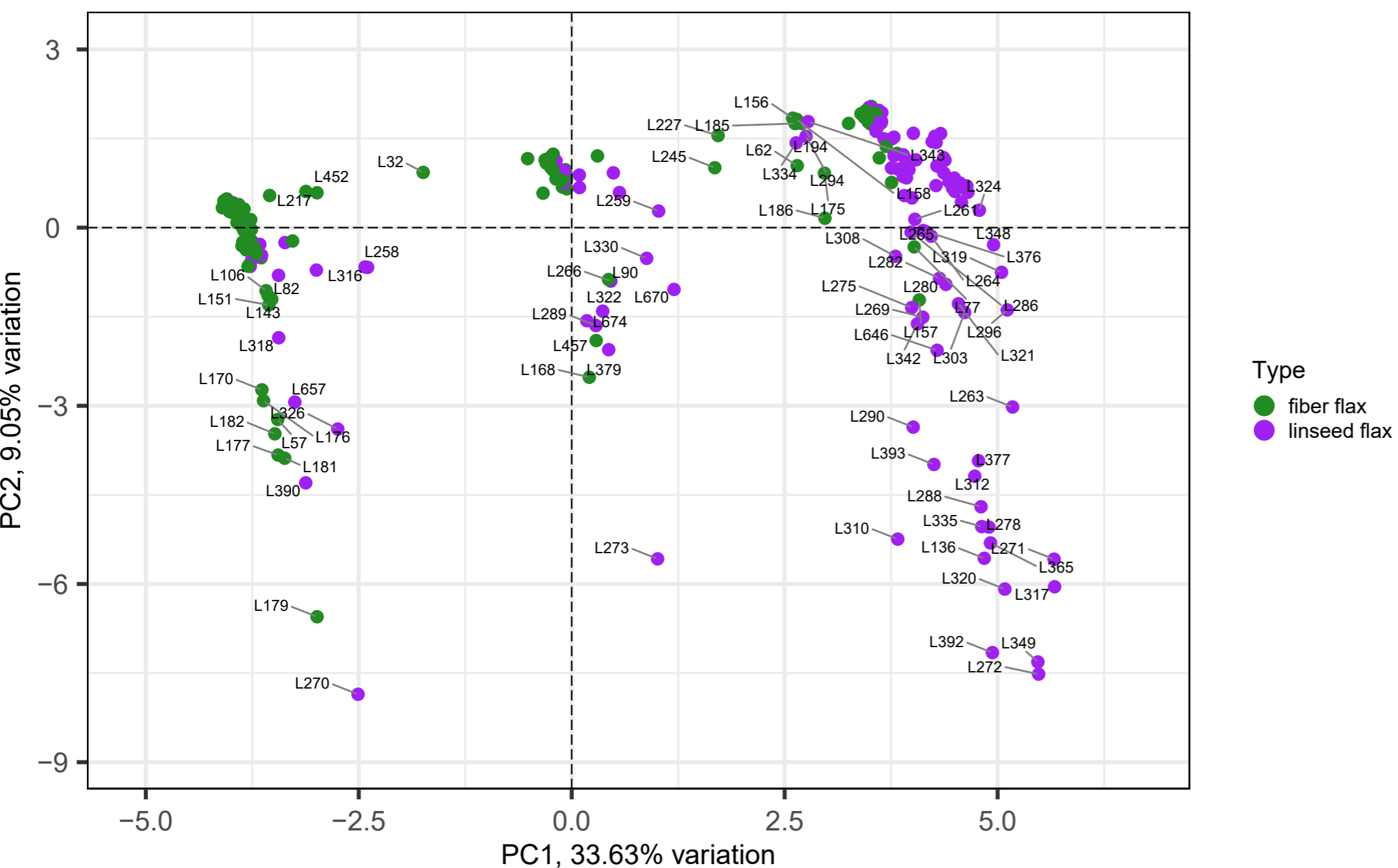

Supplement: Supplementary file 1 [file ijms-22-12383-s001.zip › 20211110_FigureS1.pdf]

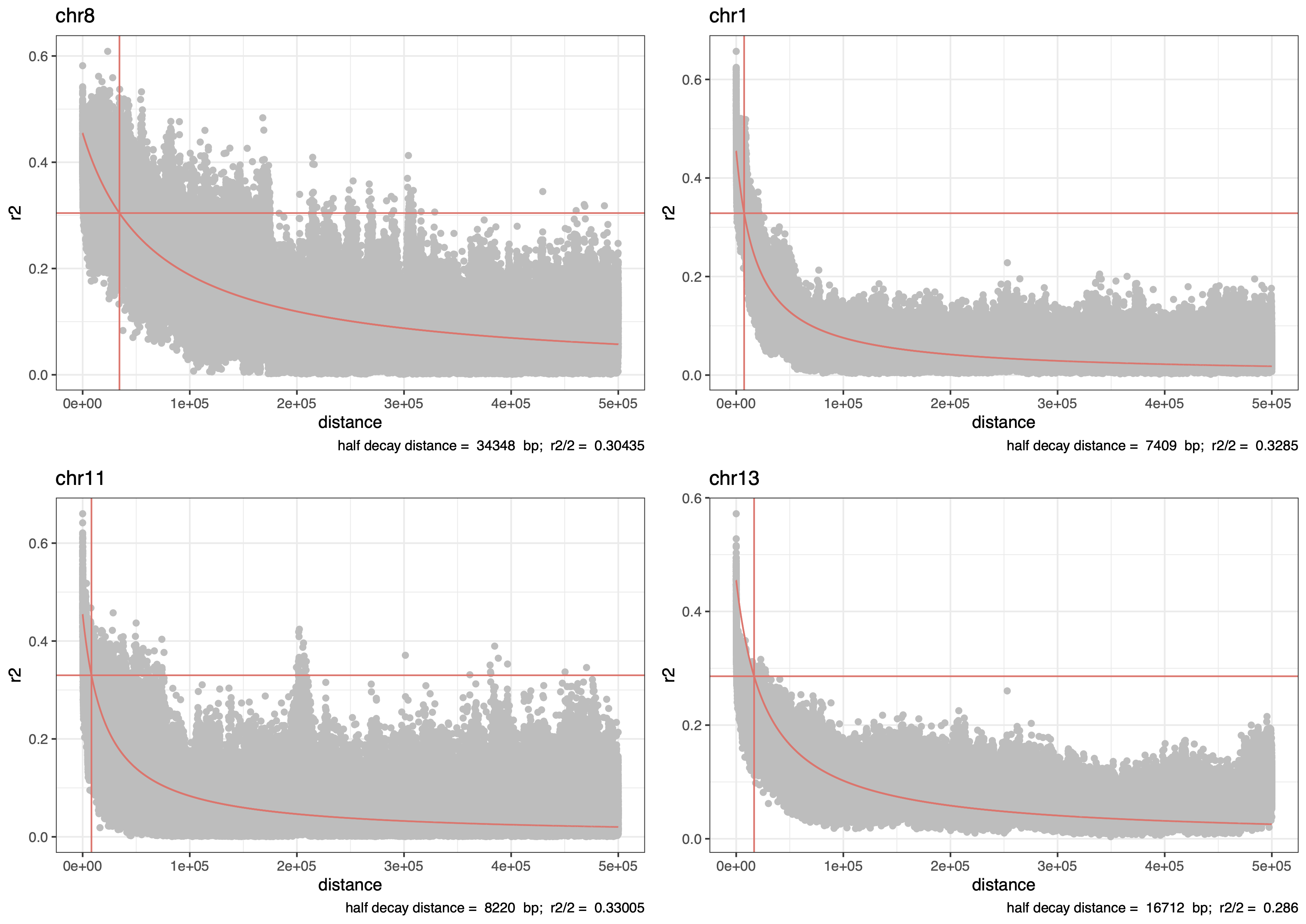

Supplement: Supplementary file 1 [file ijms-22-12383-s001.zip › 20211113_FigureS4.tiff]

Q-normalized DSI 2020

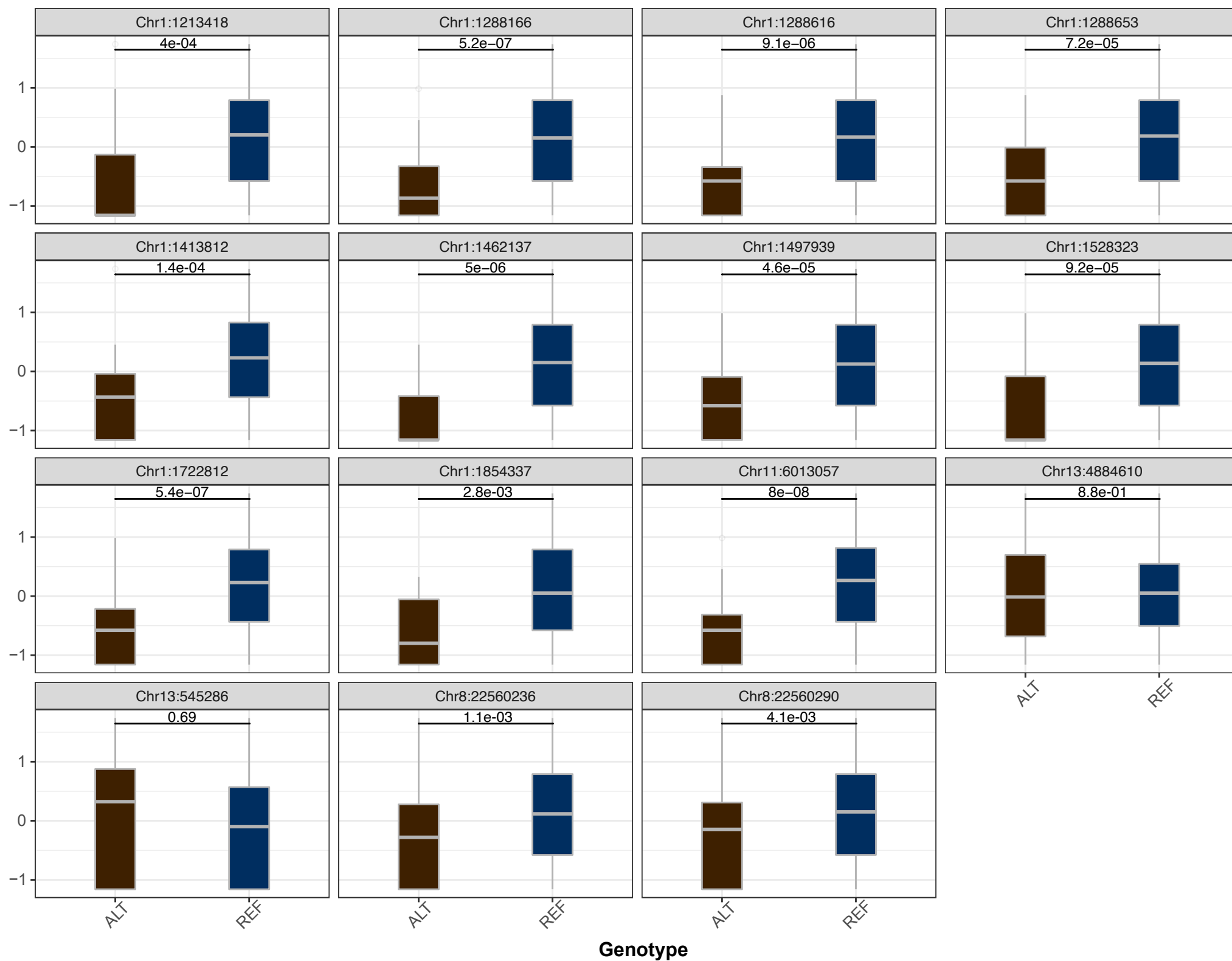

Supplement: Supplementary file 1 [file ijms-22-12383-s001.zip › 20211116_FigureS2.pdf]

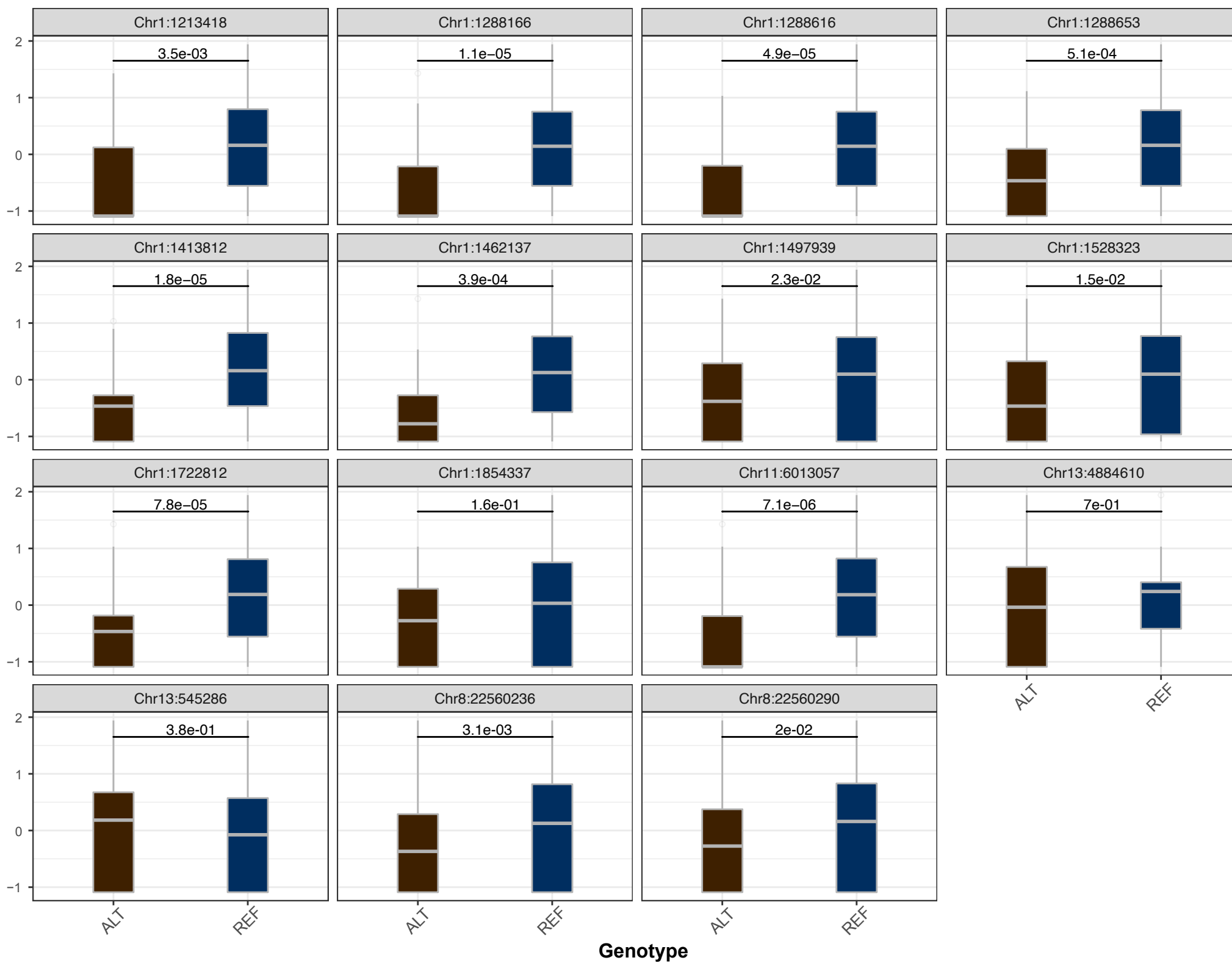

Supplement: Supplementary file 1 [file ijms-22-12383-s001.zip › 20211116_FigureS3.pdf]
